# The Role of Second-Person Information in the Development of Social Understanding

**DOI:** 10.3389/fpsyg.2017.01667

**Published:** 2017-09-25

**Authors:** Chris Moore, John Barresi

**Affiliations:** Department of Psychology and Neuroscience, Dalhousie University, Halifax NS, Canada

**Keywords:** development, interaction, intentionality, intentional relations, social understanding, second-person

## Abstract

We consider the second-person or interactive approach to social understanding, conceived as an understanding of intentional relations. We identify five forms of second-person information – self-directedness, contingency, reciprocity, affective engagement, and shared intentions – that occur only in interactions. We assess the extent to which these forms of information are available to observers of interactions as well as to the participants of an interaction and conclude that whereas observers may gain some second-person information, interactive participants have a privileged position. We also ask whether these forms of second-person information can deliver social understanding in terms of the understanding of intentional relations that are descriptive of persons. We argue that whereas none of these forms alone is sufficient for understanding intentional relations, they all play an important role in the developmental processes that enable the construction of social understanding. Therefore, the second-person approach, understood as theorizing how second-person information available in interactions is used in the development of social understanding, is a critically important approach to a full theory of social understanding.

## Introduction

Theory and research on social understanding has for many years been dominated by an orientation that prioritizes first- and third-person knowledge of psychological activity. It has been assumed that social understanding has its roots in the observation of others’ (third-person) or one’s own (first-person) psychological activity. Information from either or both of these perspectives is used to make sense of the psychological activity of both others and the self, and this understanding allows the prediction of the activity of psychological agents. Furthermore, this understanding is functional in the sense that it organizes social behavior, allowing people to act in ways that are socially adaptive. Traditionally the theory theory approach (e.g., Gopnik and Wellman, 1994) has emphasized the third-person observation of the activity of the agents and the construction or maturational unfolding (e.g., Leslie, 1994) of a conceptual system that is used to explain and predict such behavior. In contrast, the simulation approach (e.g., Gordon, 1986; Harris, 1992; Gallese and Goldman, 1998) emphasizes the importance of first-person experience as a model for understanding others. Whereas these two theoretical approaches may be seen as two poles in the explanation of how psychological activity is understood, they are united by the assumption that it is the psychological activity of individual agents (self and others) that is both the source of information about, and the target of, social understanding.

The idea that social understanding has its basis in first- or third-person experience of the psychological activity of individual agents has not gone unchallenged, however, and an alternative approach has been gathering momentum particularly over the last 10 years (Gallagher, 2001; Ratcliffe, 2007; Reddy, 2008; de Jaegher, 2009; de Bruin et al., 2012; Schilbach et al., 2013). This alternative orientation encompasses various theoretical proposals, which are united by their shared idea that interactions between people provide the context and the conditions for social understanding. This general approach has variously been called interaction theory (Gallagher, 2001) or the second-person approach (Ratcliffe, 2007; Reddy, 2008; Dullstein, 2012; Schilbach et al., 2013). Whereas some theorists prefer to refer to this general approach as ‘interactivism’ (e.g., Michael, 2011), some others (e.g., Reddy, 1996, 2008) prefer the term ‘second-person’ because they believe the fundamental distinction between this approach and the theory theory or simulation approaches is the emphasis on a distinct form of experience that is available to participants within interactions but not through the observation of individuals’ activities. Since our focus here will be on novel forms of experience and social information about persons that is available in interactions that are not available in non-interactive contexts, we will use the term second-person approach in the present paper.

The second-person approach has a number of interrelated theoretical roots, including the embodiment approach to psychology (Varela et al., 1991; Gallagher, 2001; Thompson, 2001), phenomenology (Merleau-Ponty, 1964; Husserl, 1970), primary and secondary intersubjectivity (Trevarthen and Hubley, 1978; Trevarthen, 1979, 1980), and direct perception (e.g., Gallagher, 2008), whereby it is assumed that interactive or second person (Reddy, 1996) contexts provide for an immediate and qualitatively distinct form of social knowing. Various authors have reacted to the second-person approach in recent years (e.g., Michael, 2011; Herschbach, 2012; Overgaard and Michael, 2015; Schönherr, 2016), pointing out its limitations for a theory of social understanding. Whereas we follow these latter authors in the belief that the second-person approach cannot fully displace first- and third-person approaches, we also believe that there is a core of fundamental importance in the second-person approach. Our goal in this paper is to support the second-person approach by showing how the experience of interaction may yield forms of information that are unique and critical for social understanding. In the bulk of the paper we introduce in more detail the second-person approach (section “Second-Person Approaches to Social Understanding”), describe these forms of information (section “Varieties of Second-Person Information”), and then consider how they may contribute critically to the development of social understanding (section “Pulling It Together”). Before that, however, we first consider the nature of social understanding as the target of this theoretical investigation. Here we draw heavily on intentional relations theory (Barresi and Moore, 1996).

### The Nature of Social Understanding

At the outset it is important to be explicit on the explanandum for this theoretical enterprise. What are we ultimately trying to explain by appeal to the psychological processing of first-, second-, or third-person forms of experience? Since 1978 (Premack and Woodruff, 1978), the most frequent characterization of social understanding has been in terms of mentalizing or ‘theory of mind.’ However, as a result of dissatisfaction with the restrictive nature of the characterization of social understanding as ‘mental,’ which tends to presuppose a form of mind-body dualism, many authors have sought alternative characterizations (e.g., Carpendale and Lewis, 2004; Tomasello et al., 2005; Moore, 2006; Reddy, 2008). Here we adopt the non-dualist account that we have previously provided in a variety of publications since 1996 (e.g., Barresi and Moore, 1996; Moore, 2006). Our account of social understanding is broadly compatible with many others but has certain core features which we believe should be emphasized. First, we take social understanding to be about understanding intentional relations (Barresi and Moore, 1996). By ‘intentional relations,’ we mean the variety of forms of object- or goal-directed psychological activity in which agents engage. Intentional relations always involve three components – an agent, an object that is the focus of the psychological activity, and a relation linking them. In this conceptualization, the term ‘intentional’ subsumes all object-oriented activity. It thereby includes but is not limited to purposeful action. It is intentional in the sense of ‘aboutness’ that goes back to the formulation by Brentano (1874/1973), who described ‘intentional inexistence’ as the essential characteristic of mental states. However, our view of intentional relations has more in common with that concept as used by phenomenologists, in particular with the late views of Merleau-Ponty (1964, 2012) and Husserl (1989), where the aboutness relation has its foundation in activities of embodied subjects that are directed toward other objects, including other embodied subjects, that exist with them in a common world (cf. Zahavi, 2001; Barresi, 2008). Where we differ from the phenomenological approach is in not attempting to lay a constitutive foundation for intentionality in subjectivity or consciousness, or in an *a priori* form of “intersubjectivity” (Zahavi, 2001). Rather, our approach is to assume that there are objective psychological or ‘intentional’ relations that occur between embodied agents and other objects, including other embodied agents with whom they share “common worlds” (Barresi, 2004, 2007, 2008). These intentional relations come to be recognized and understood by humans in a form of “common sense” psychology (Moore, 2006). Such a conceptualization of psychological activity is now widely accepted among researchers (e.g., Tomasello et al., 2005; Woodward, 2005; Iacoboni et al., 2007; Pacherie, 2008; Musholt, 2015).

Second, although social understanding may recognize the intentional activity of a range of agents including non-human ones (e.g., other animals, certain autonomous non-biological agents, such as robots), when it comes to humans, agents are assumed to be persons (Strawson, 1959; Barresi et al., 2013). By this we mean that human agents form a category of ‘persons’ that includes other people and the self and conceives a categorical equivalence between others and the self. Each of us recognizes ourselves to be persons like others and we recognize others to be selves like ourselves.

Third, the relational links between agents and objects are of different kinds, but generally fall into three main categories or modes. Epistemic relations involve informational links, which may be sensory, perceptual or cognitive (e.g., seeing and believing). Conative relations involve motivated or goal-directed action and practical intentional attitudes (e.g., desires and prior intentions). Affective relations involve emotional orientations or attitudes (e.g., loves and fears).

Finally, while some of the objects of intentional relations are purely imaginary or representational, and may be said to have intentional ‘content,’ most involve enactive relations to real objects and individuals, thus, are fully embodied psycho-physical relational states. In this way, intentional relations subsume psychological orientations both to actual physical objects and to mental objects.

### The Limits of First- and Third-Person Approaches to Social Understanding

The two traditional theories of social understanding – simulation theory and theory theory – have tended to prioritize either first- or third-person forms of information as the basis for social understanding. The fundamental difference between first- and third-person information about intentional activity is that first-person information pertains to an actor’s own intentional relations while third-person information pertains to the intentional activity of another person. We have argued in other places (Barresi and Moore, 1996) that such theories that emphasize either first- or third-person information provide a limited basis for understanding intentional relations as the object-directed activities of persons. To see why, it is necessary to consider in more detail in what first- and third-person information consist.

First-person information is the kind of information that is available to an actor about their own intentional activity. In Barresi and Moore (1996), we claimed that such information in its base form, i.e., when uncontaminated by more mature forms of social understanding, is primarily information about the intentional objects toward which the agent directs his or her activity. For example, when reaching for a desired object, such as a cookie, the actor in the grip of the desire is aware of the goal cookie and perhaps the desire, but is not immediately aware of themselves as the desirer. As such, first-person information is primarily about the objects or goals of intentional relations as well as about the actor’s intentional orientation to those objects. Although first-person information is information about the self’s intentional activity, it is not about the self as an intentional agent; rather, it is the experience of self when one is engaged in intentional activity directed at other objects. Recently, Musholt (2015) has provided a clear and detailed articulation of why the self is only implicit in first-person information and how representations of first-person information of intentional relations are fundamentally ‘self-less.’ Therefore, first-person experience alone does not deliver the appropriate information to represent self as an intentional agent or person.

In contrast, third-person information is the kind of information available to an observer of another person’s intentional activity. In its uncontaminated base form, it is primarily information about the actor and his or her action, but may contain little or no content about the actor’s intentional object, particularly if the object is at a distance or representational. For example, if one passively observes another person turn their head and gaze into the distance, we are directly aware of the actor and the act of turning and looking, but we are not directly aware of the object of their attention. In order to gain the latter information, we would also have to turn and look in the same direction and realize that the object we now perceive is the object of the other person’s attention. Therefore, third-person experience alone cannot deliver appropriate information about the object toward which the other person’s intentional action is directed.

We have argued that the fundamental and qualitative distinctness of first- and third-person information will prevent a full understanding of intentional relations without some mechanism for bridging the gap between them (Barresi and Moore, 1996). Without such a mechanism, the concept of intentional relations involving persons or selves with both first- and third-person characteristics will remain elusive. In our earlier work (Barresi and Moore, 1996), we referred to this mechanism as the ‘intentional schema’ that combines first- and third-person information into a common representational format that can be applied to either first- or third-person forms of information. We suggested that participation in interactions in which the intentional relations of self and other were matched provides the contexts in which the intentional schema can operate to form concepts of particular intentional relations. However, as certain commentators on our earlier target article argued (e.g., Gomez, 1996; Reddy, 1996), in our discussion of interactions we paid rather less attention to the unique type of experience available within them that forms the basis for the second-person approach. It is to that experience that we now turn.

## Second-Person Approaches to Social Understanding

The fundamental insight of second-person approaches is that social understanding depends critically on interactive engagement with others. These approaches stand in contrast, therefore, to the traditional accounts of social understanding, which posit that observation of the individual activity of other agents (e.g., theory theory) or of the intentional activities of the self (e.g., simulation theory) can provide the representations necessary for inferring, understanding, and predicting the behavior of agents. Second-person approaches have theoretical roots in a combination of the embodiment approach to cognition (e.g., Thompson, 2001) and of the phenomenological approach to intersubjectivity (e.g., Gallagher, 2001). These approaches are also linked to intersubjectivity and relational approaches to psychological development such as those of Trevarthen (1979), Hobson (2002), and Reddy (2008). The embodied cognition approach to psychology grew from a reaction to the abstract information-processing and computational approaches to mind but lends itself very well to those who argue that social understanding depends fundamentally on interaction. According to the embodied cognition approach it is essential to view cognition as situated in, and guiding, real world activity. Furthermore, the mind should not be seen as dissociated from the environment but embedded within it, such that the scientific study of cognition must take as its object the mind/body-world complex.

When the embodied cognition approach is applied to social cognition, it is clear that the environment and the activity for which social cognition is organized is the social world. Because typically social actions are performed in relation to other people, who themselves are performing responsive actions, social actions are normally embedded within social interactions. Thus, it makes sense that social understanding is grounded in and depends fundamentally on interactive embodied social processes.

It is important to recognize that within the embodiment approach embodied action is sensorimotor. That is, it involves perception of the world and its objects just as much as motor action in relation to the world and its objects. Within this view, perception of the social action of others is posited to be direct (Gallagher, 2008) so that the information available through perception directly delivers information about social affordance. Second-person theorists have claimed that such direct perception includes information about intentional relations (Gallagher, 2008). Therefore, understanding of intentionality does not have to be inferred from information that is non-intentional in nature. These second-person theorists claim that direct perception of the other person’s actions is all that is required for mutual understanding in social interactions.

But the capacity for ‘direct perception’ of information indicating intentional activity of another person does not mean that this information immediately delivers intentional understanding. If understanding intentional relations in an agent-independent format requires integration of first- and third-person information through an intentional schema, then what is directly perceived by social organisms may be limited to what Barresi and Moore theorize as only third-person information of intentional activity of others, and first-person information for self. Typically, this is all that is required for the coordinated and co-regulated interaction between two actors that can be interpreted as mutual understanding. So, for instance, socially appropriate responses of virtually all social animals need not rely on understanding the activities of others as involving an agent, psychological relation, and an object. All that is required is to perceive directly the actions and expressions of other agents, and the apparent directions of these actions, to determine their social affordance for self. Being a participant in multiple interactions over time might provide this kind of know-how without providing an integrated conceptual understanding of intentional relations that can be applied uniformly to self and other. A non-conceptual understanding of how to act in response to various directly perceived expressions and actions of others is sufficient. Non-participants cannot experience this know-how because of its practical nature. At best, they might imagine themselves in the situation and ‘know how’ they would react, but this is not the same as categorizing that reaction in terms of intentional concepts that can be applied uniformly to self and other. Even so, it may be that it is only through social interaction that one may come to an integrated understanding of intentional concepts of this sort.^1^

At first blush, there may seem to be something of a disconnect between the claim that social understanding of intentional activity must take an embodied approach and the requirement for interactive experience. Clearly, the intentional activity of both self and others may occur outside of interaction, but surely that does not mean that such activity cannot be known in terms of a non-dualist approach to embodied intentional relations. While alone, I may reach for a cup of tea on my desk and understand that action in terms of my goal-oriented desire for the tea. Similarly, the claim of direct social perception may be applied just as much to third-person, non-interactive, and observation. So, while casually looking out of the window at the café I may observe someone reach down and pick up something off the sidewalk and immediately understand that action as their interest in, or desire for that object. Indeed, the capacity for a kind of direct, non-interactive social understanding has been shown to occur even for infants observing the goal-directed actions of others (Woodward, 1998). Furthermore, one must recognize that a substantial amount of cognitive activity clearly goes on decoupled from the immediate environment – e.g., planning, remembering, counterfactual thinking, and, while these activities are more purely mental in not immediately being expressed in behavior, they are nevertheless accessible as experiences in self, and connected to earlier or later actions in others.

Having considered the theoretical roots of the second-person approaches, we believe it is critical to distinguish two issues in connection to these approaches. First, one of the core criticisms raised by certain second-person theorists about theory theory and simulation approaches to social understanding is that those approaches take a spectatorial stance (e.g., Gallagher, 2001; Thompson, 2001; Hutto, 2004; Reddy, 2008; Schilbach et al., 2013). A spectatorial stance means that the person attempts to make sense of people’s activity by adopting a passive observational stance (e.g., de Bruin et al., 2012). In this sense, human activity is an object to be understood by observation and explanation, much like one would for any object or event, such as an earthworm or an apple falling from a tree. Such a spectatorial approach can in principle be applied to intentional activity, whether of the self or others, though it is generally focussed on the activity of others. Second-person theorists claim that such a spectatorial stance misses the primary manner in which people come to know others, that is through interaction or second-person engagement – experiencing the other as a ‘you’ to one’s own ‘I’ (Reddy, 2008). Therefore, the second-person approach argues that a passive spectatorial stance provides a different kind of experience than that arising out of action in relation to, and in interaction with, others. So the second-person approach to some extent contrasts with first- and third-person approaches in that it is participatory rather than spectatorial.

We believe, however, that this type of concern over spectatorial rather than participatory experience is not the fundamental issue (see also de Bruin et al., 2012). Agents might participate in interaction with others and still adopt an observational stance in the sense that they attend to their own intentional activity and to that of the other while in the interaction (e.g., Barresi and Moore, 1996). Even as participants, they would then have access to both first- and third-person information about the intertwined intentional relations of self and other, respectively. However, the first- and third-person information would still be of qualitatively different kinds, and so alone could not yield a form of social understanding of intentional relations common to both self and other. So, interactions will present first- and third-person information about the activities of the agents involved in the interaction. But, the fact of being a participant in an interaction does not obviate the need for an additional component to bridge the gap between self and other for a uniform understanding of intentional relations.

In Barresi and Moore (1996), we postulated that the missing component for bridging the gap between self and other was an intermodal ‘intentional schema’ that developed through participation in matched interactive activity directed at a common object, such as occurs in joint attention or imitation. For example, when an infant becomes capable of following the gaze of another person in the second half of the first year, she experiences in such joint attention episodes the third-person information of the other looking at the object and the first-person information of her own attentional shift and visual apprehension of the object. Such combined experiences might allow the construction of representations of joint activity that capture both first- and third-person qualities of the activity and thus can be equally applied to self and other. We saw this as a crucial stage on the way toward full cognitive understanding of intentional relations. Our suggestion was rejected by early proponents of the second-person account (e.g., Reddy, 1996, 2008) for being spectatorial rather than participatory, and insofar as it relied only on first- and third-person information of interactions it is. But it is important to note that it was nevertheless an account that was fundamentally dependent on interactive processes, and therefore participatory in that sense. The limitation of our account was that it focussed only on first and third-person forms of information that were available in interactions.

The second, and we believe more important, aspect of the second-person approach with respect to conceptual understanding intentional relations is the claim that within interactions there is a fundamentally different kind of information presented than that available from observing one’s own or another person’s individual intentional activity. One does not have to disavow information-processing theory in order to appreciate that within interaction, each agent’s activity is connected to the activity of the other and that this connection provides a form of dependence or correlative information that is qualitatively distinct from first- or third-person information alone. Second-person information is irreducible to first- or third-person information because it inherently incorporates information from both the self’s and the other’s psychological activity. Thus, when in interaction, each actor has information about their own activity (first-person), information about the action of the other (third-person), and information about the interconnection or interdependence of the activities of both (second-person). All three forms of information feed into the cognitive process of understanding intentional relations.

In what follows, we pursue this line of analysis and we discuss in detail the forms of information that are only available in interactions. But, even here, it is important to distinguish between second-person information that occurs in an interaction, and being one of the participants of the interaction. It may be that the second-person information that arises in an interaction is equally useful to competent observers of, as well as actors in, the interaction. However, it is also possible, and congruent with the emphasis of second-person theorists, that participants have a special advantage in processing the second-person information that they directly experience and helped generate, and that this information combined with first- and third-person information is crucially important in social cognitive development. We will first identify the kinds of second-person information that occur in interactions. We will then argue that those forms which are merely dyadic and do not include another object, lay the foundational substrate for understanding intentional relations, but that only toward the end of the first year, when infants enter into triadic interactions which include other objects, is it possible for them to begin to acquire an understanding of intentional relations in a uniform fashion across individuals, including self and others.

## Varieties of Second-Person Information

Various claims for the characteristics of second-person information exist in the literature. Here we examine five – self-directedness, contingency, reciprocity, affective engagement, and shared intentional relations. All of these characteristics have been proposed as significant aspects of second-person information, although different theorists tend to see different degrees of importance in each of them for social understanding and rarely, if ever, are the distinctions among them clearly articulated. As we review the five types of second-person information, we will also consider the extent to which they are available to an observer outside the interaction (hence non-participatory or purely spectatorial) as well as to the participants within the interaction. For, if these forms of information are just as evident to an observer of an interaction as to a participant in that interaction, then it is not clear what advantage actually participating in interactions will bestow as far as understanding intentional relations goes. As we shall see, certain aspects of second-person information may well be to some extent evident to an outside observer, whereas others may be less evident.

### Self-directedness

By self-directedness, we mean that within interactions each agent gains a particular perspective on the activity of the other which indicates in a unique way that the latter’s activity is at least in part directed at the former. For example, if I am engaged in a face-to-face discussion with another person, that person’s actions are directed toward me, e.g., her body, limbs, face, and eyes are oriented toward me. This perspective is different from that gained by observing another when not in interaction. The experience of self-directedness distinguishes interaction from non-interactive observation of intentional relations. Inevitably, self-directedness as a form of information is not available to an outside observer of an interaction; it is premised on participation.

In support of the psychological validity of this distinction, it has been shown that adults process self-directed social information differently from social information that is not self-directed (e.g., Senju and Johnson, 2009; Schilbach et al., 2013). Although it has been suggested that such differential processing depends on relatively high level intentional attribution (Myllyneva and Hietanen, 2015), other evidence implies this kind of discrimination has deep developmental roots (Reddy, 2003). Even shortly after birth, infants look longer at faces with direct eye gaze compared to faces with eyes averted (Farroni et al., 2002) and neurophysiological evidence of this discrimination has been shown within the first few months of life (e.g., Grossmann et al., 2007).

Discriminating self-directedness information from non-self-directed information is not simply perceptual in nature. There are also affective implications of self-directedness information (Reddy, 2008). For example, different affective reactions occur to direct versus averted gaze. Hietanen et al. (2008) showed heightened emotional arousal in participants viewing the direct gaze of real people compared to averted gaze, although the same effect was not shown for photographs. Furthermore, direct gaze enhances processing of approach-oriented emotions such as anger and joy, whereas averted gaze enhances processing of avoidance-oriented emotion, such as fear and sadness (Adams and Kleck, 2005).

What role might self-directedness information play in the understanding of intentional relations? Some have argued that self-directedness implicitly or explicitly establishes the self as a focus of the other’s activity and that this aspect of second-person information provides the primordial information for the development of a concept of self as an objective entity (e.g., Reddy, 2003). The idea that the concept of self is rooted in the actions and attitudes of others toward the self has a long history in psychology, with roots in the social psychology of authors such as Baldwin (1894/1898), Mead (1934), and Cooley (1992). However, we are skeptical of the claim that self-directedness is the original source of information about the self. Insofar as this kind of second-person information is primarily information about the activity of another person, not about the interaction, it might be better conceptualized as a particular form of third-person information. As such, like third-person information gained from the observation of agents engaged in any activity, whether interactive or not, it does not necessarily include directly information about the object of the other’s activity, though it may serve as a basis for discriminating one object from another. Because it is information that is peculiarly available when in an interaction, we, like others (e.g., Schilbach et al., 2013), class it as second-person. But that does not mean that the self-directedness necessarily carries implicitly or otherwise information about the self, particularly as a subject and agent of intentional relations. The self-directedness of early interactions where the focus of the interaction is the body parts of the infant may constitute the original interactions over shared objects, and, as such, may play a role in forming a concept of an objective self. However, being an object of someone’s attention is an integral ingredient in any form of second-person interaction even when one’s bodily self is not a shared object of intentional relations. All the other forms of second-person information that we will now consider depend for their existence on mutual attention of this sort between two agents. So, insofar as second-person information is necessary for any form of social understanding to occur, this form of information is the primordial ground upon which a conception of self and other as persons and selves engaged in intentional activity is eventually built. But this is not because on its own it delivers direct knowledge of self. Rather, it is because, when combined with knowledge gained of the intentional activity of both self and other in shared object-directed contexts, it provides a ground for understanding both self and other as persons and selves.

### Contingency

By contingency, we refer to the temporal coordination of information within interaction. In interaction, because the activity of each agent tends to be in response to the activity of the other, the intentional relations of the participants tend to form reliable temporal relations to each other. There is therefore temporally contingent information about the connection between the activities of both agents. From the point of view of each agent, there is temporally based information about how one’s own action predicts the action of the other, and vice versa. Interactions typically involve actions of both participants sequenced in a turn-taking fashion. Thus, if I perform an action directed in some way at you, I can expect that you will respond with an action directed at me. And if I experience an action of yours directed in some way at me, then I will tend to respond with an action directed at you. Clearly such action pairs can themselves also be sequenced temporally to allow long chains of interaction. Understood in this way, contingency refers only to the temporal patterning of actions in time, such that the temporal parameters are regular and, at least in direct physical interaction, relatively short. Indeed, in synchronized actions the movements of several participants of an interaction approximate simultaneity.

It is important to recognize that contingency is also present in first-person information in the sense that first-person information about one’s goal-oriented action is contingently tied to information about the effects of that action in the world. Indeed, it has been argued that such contingency information is the critical information to detect one’s own agency (e.g., Russell, 1996; Longo and Haggard, 2012). Observed events that are the result of one’s own action are tied contingently to the first-person information generated by action, whereas events that are not so caused tend to be uncontingent in relation to action. How does this contingency information differ from second-person contingency? Here it is critical to distinguish between the perfect contingency that is present in first-person information about the self’s intentional activity and the imperfect contingency that exists in second-person information when in interaction with others (e.g., Watson, 1979; Bigelow, 1999). Even while maintaining an interaction through mutual attention to each other, the other person will respond some of the time but not all of the time and so the probability of a contingently tied response to the self’s action is somewhat less than that afforded by sensory-motor contingency and direct causal contact with the non-social world. It is this imperfect contingency information about extended sequences of interaction between agents that is considered second-person because it is only available when agents are in interaction.

Interestingly, however, some degree of second-person contingency information may well be evident to an outside observer of the interaction. An observer of an interaction between two other agents may still be able to observe the temporal patterning of the interactants’ actions within the interaction (Bassili, 1976). So, whereas contingency information is a property of interactions, it is not necessarily only apparent to the participants in the interaction. However, when the temporal information of contingency is combined with the self-directedness information present within interaction, it is clear that again this particular complex of contingent self-directedness is special to the participants within the interaction.

Various authors have argued for the importance of imperfect contingency information in social understanding (see especially Watson, 1979, 1985; also Bigelow, 1999). A classic illustration dates to Watson (1972) who showed that infants as young as 2 months can detect contingency in instrumental learning and that when they do they exhibit pleasure. Noticing the similarity between the enjoyment expressed in the discovery of the contingency of instrumental learning and that expressed in contingent social interactions, Watson argued that infants learn about and come to enjoy social interactions because of their contingent properties (Watson, 1972; see also Stern, 1985 in Michael, 2011). But he stressed that optimal interest and pleasure is obtained when the contingency information is less than perfect.

Later research with young infants showed that infants become distressed when contingency information in interaction is removed completely (e.g., Tronick et al., 1978; Murray and Trevarthen, 1986). For example, in the still-face procedure, mother and infant interact normally for a period before the mother adopts a still, expressionless face for a short period. This period is then followed by a return to normal interaction. The still-face procedure has been shown reliably to disrupt infant emotional state and typically leads to distress and loss of behavioral organization (see Mesman et al., 2009). In contrast to the still-face approach which removes all socially contingent stimulation, Murray and Trevarthen (1986) attempted to retain all of the maternal infant-directed behaviors while disrupting contingency by allowing infants to interact with a prerecorded video of maternal behavior. In this case, infants still became distressed in comparison to a presentation where they were able to interact with a live video of the mother, who also could see them. Although, this finding has led many authors to conclude that infants are social attuned (e.g., Reddy, 2008), as Gergely and Watson (1999) argue, this study at best demonstrates that infants detect a lack of contingency in interaction and are perturbed by it. However, the Murray and Trevarthen (1986) study remains ambiguous,^2^ because although the recorded maternal behavior presents the same kinds of behavior as in a normal interaction, those behaviors lack not only the temporal coordination of contingency but also a meaningful connection to the infant’s.

### Relevance and Reciprocity

Consideration of the connection between the actions of each participant in an interaction also requires attention to the characteristics of those actions. Not only is the activity of each agent within an interaction linked in time to the activity of the other, it is also linked in form. We need to expand the consideration of the second-person properties of interaction beyond mere temporal contingency to include the nature or form of the activity. The nature of one agent’s action will constrain the nature of the interactive partner’s action. If I indicate to you a colorful bird in a tree, you are likely to respond by looking and commenting on it, not by scratching your armpit. And, when you do respond appropriately, then I will acknowledge in some way your response. Another way of putting this is to say that within an interaction, each participant’s action is not only linked in time but it also involves relevance and reciprocity. Interactions are events in which the action of one participant leads to a relevant reaction from the other, which in turn leads to a further relevant reaction from the first. The temporal sequence of mutually relevant reactions constitutes reciprocity. Without doubt, at least for those interactions beyond early infancy, participants’ expectations of the other’s action within interactions include reciprocity.

What defines whether a reaction to one’s action is deemed relevant and reciprocal? A rich definition (e.g., de Bruin et al., 2012) would include the notion of common ground, a shared topic and perhaps new information, and we will examine these ideas later. For now, however, we have in mind a simpler definition that more minimally establishes reciprocity as enabling each participant’s goals in the interaction to be attained and therefore allowing the interaction to continue. If one participant responds to the other in a way that enables the latter in turn to respond again, then both participants’ responses may be said to be relevant. In this way, reciprocity is identified as a property of any ongoing interaction and one might reasonably extend it to cover non-human interactions or human interactions that are purely dyadic in form, for example, the first interactions between infant and mother (Brazelton et al., 1974). Indeed, even in an antagonistic interaction, each interactant’s action may be said to be reciprocal to the other’s. So, reciprocity refers to the fact that the activities of self and other within an interaction are not only temporally coordinated but also relevant to each other. Interactions are events in which the action of one participant leads to a particular relevant reaction from the partner, which in turn leads to a further relevant reaction from the first, and all the while this mutual dependence or reciprocity serves to maintain the interaction.

Some authors have claimed that reciprocity is the core of second-person information. In particular, de Bruin et al. (2012, p. 5) write, “what distinguishes 2P [second-person] from 3P [third-person] modes of social cognition is their reciprocal nature,” but it is important to recognize that for them reciprocity is assumed to depend on shared objects or common goals. They go on to add, “reciprocal interaction depends on the ability to share representations of objects and events with others” (p. 5). In contrast, we view reciprocity as more basic than this. As we shall see later in section “Shared Intentional Relations,” reciprocity includes interactions with shared representations and common goals, but it is a more general category of second-person information that admits dyadic interactions with no shared objects or goals.

Reciprocity in interactions provides an additional form of second-person information that is not available in individual activity; it depends absolutely on the nature of interactions. Interactants are aware of this form of information; apt relevance of the other’s activity to one’s own activity is a key aspect of maintaining an interaction for both participants. To the extent that the other’s activity is not relevant and does not sustain reciprocity, the interaction can be said to be failing and is likely to be discontinued by either or both participants. It is unclear, however, to what extent a non-participant observer of an interaction has access to this aspect of second-person information. Certainly, some of it will be available to any observer. That two interacting participants are responding reciprocally to each other’s activity can be detected to some extent by an outside observer. Indeed, it is possible that if the observer knows either or both of the interactants well, a good deal of the relevance information will be evident. However, we suggest that an observer will almost always be at a disadvantage with respect to detection of reciprocity compared to the participants in the interaction who will be aware of the extent to which each participant’s activity meets their respective goals for the interaction.

The next two categories of second-person information may be considered to be subcategories or specializations of relevance and reciprocity. However, we distinguish them here because they are argued to be of special importance for social understanding.

### Affective Engagement

Interactions, whether pro- or anti-social, are often imbued with considerable affective content and this aspect of interaction has commonly been taken by second-person theorists to be a significant component of the experience of prosocial interactions (Schilbach et al., 2013). Within prosocial interactions, there is typically a degree of affective engagement, so that both participants are emotionally invested in the interaction. Affective engagement means that within prosocial interaction, there is a mutual motivation to maintain the interaction, typically because it is enjoyable to both participants. In such cases, each participant responds with positive affect to the observed positive affect of the other. Affective engagement may be seen as a particular form of reciprocity in that affective intentional relations of one participant are related in form to the affective intentional relations of the other participant. However, like other theorists (Schilbach et al., 2013), we consider affective engagement separately because it is critical to maintain the interaction. Without affective engagement, the likelihood of an interaction proceeding is severely reduced. So, it is not simply the case that each agent’s actions have relevance to the other’s, each agent shares a motivation to maintain the interaction and gains satisfaction from doing so. In addition, because of natural empathic processes (Zahavi, 2015), affective engagement may involve a form of resonance that creates a mutual modulation of affect, including amplification or depression of affect. For example, each participant responds to the other’s smiling as an expression of positive affect with a compatible expression so that the experience for both is of a heightened affective arousal. Of course, it would be inaccurate to claim that all interactions are characterized by mutually directed positive affect. We have all had the experience of a ‘difficult conversation’ – an interaction that needs to take place despite it being aversive to all concerned. Nevertheless, it is the case that for any prosocial interaction to proceed there must be a motivation to engage and then the engagement is characterized by affective meshing of the actions of the participants.

Whereas aspects of information about affective intentional relations may be evident in first- and third-person information alone, affective engagement is a form of information that is specific to interactions because the affective experience of each participant depends upon that of the other. In particular, the mutual affective modulation that occurs in interactions is particularly salient to those directly involved. Outside observers may be able to detect aspects of the engagement in the same way as for aspects of contingency and reciprocity, however, the particular experience of affective engagement is unique to being a participant.

For some authors (e.g., Hobson, 2002; Reddy, 2008), affective engagement in interactions is the most developmentally primitive and therefore fundamental form of second-person experience [although see Watson (1972), who argues that contingency detection is more primitive]. We would note that, as with contingency information, affective engagement would not be possible without the self-directedness within mutual attention that is necessary for all second-person interactions. Nevertheless, the earliest mother-infant interactions, evident from as young as 2 months of age, tend to be highly mutually affectively arousing. Various developmental theorists have argued that the affective properties of these early interactions are critical for the maintenance of these interactions (Brazelton et al., 1974; Trevarthen, 1980; Stern, 1985; Reddy, 2008). From a developmental point of view, the affective mirroring that is established in affective engagement between infants and mothers has been suggested to be a critical component for the development of the awareness of affect (Stern, 1985; Reddy, 2008). For example, Gergely and Watson (1999) propose a social-biofeedback model whereby very young infants gain an awareness of their own affective states by having them reflected to them by their caregivers in interaction. By the end of the first year, infants are able to use the affective intentional relations of others toward external objects to guide their own subsequent affective relations to those same objects (e.g., Feinman, 1982).

Notwithstanding the importance of affective engagement for the understanding of affect in both self and others, it seems clear that affective engagement alone will be of limited value for a comprehensive understanding of intentional relations. As we noted earlier intentional relations come in three broad kinds – affective, epistemic, and conative. While many forms of intentional activity may involve a combination of two or three kinds – for example, the joy in spotting one’s loved one come through the arrivals gate at the airport – others may be much more limited in flavor. It is not clear what affective engagement alone can do for the understanding of predominantly epistemic and conative intentional relations. Rather as we argue in section “Pulling It Together,” understanding of epistemic and conative intentional relations depends on epistemic and conative engagement with others.

### Shared Intentional Relations

There is another class of reciprocal interaction that plays a particularly important role in the provision of information important for social understanding and so we differentiate and highlight it here. For some interactions, reciprocity is established by the tendency to align with the object-directed action of the interactive partner. By ‘align’ we mean here that each participant in the interaction takes the same object as the focus of their intentional activity. Take the example mentioned earlier whereby one actor indicates a colorful bird in a tree. A relevant response by the interactive partner entails also attending to the same bird and perhaps commenting on it. In this case the interactive exchange involves both participants aligning their intentional relations – both attend to the same object. Such cases of ‘joint attention’ underlie most interaction in the sense that there is almost always a shared topic of interactive engagement, whether that topic is a real physically present object (such as the bird), or an abstract, represented topic (such as the ideas in this paper). So, joint attention is a form of relevancy established through the sharing of epistemic intentional relations, such as visual attention or thinking.

Reciprocal interactions involving affective and conative intentional relations may also have a shared basis in objects. Two interactive partners sampling the same new dish at a restaurant may both exhibit an expression of disgust, which is simultaneously a reaction to the food and an affirmation of the other’s culinary taste. Two movers may collaborate to lift a couch up a flight of stairs, both simultaneously reacting to the other’s movements in relation to the task at hand, a common goal for their activity (cf. Butterfill, 2012). In both of these examples, each participant in the interaction has information about their own and the other’s intentional relation and how they relate to each other. This information is intrinsically second-person.

In some activities, most obviously imitation, the intentional actions of two agents are matched; so the form of the action is the same for both participants. But this matching may not be dependent on reciprocal processes. In imitation, one actor copies the form of some goal-directed action of the other and so enters into an alignment of conative intentional relations. Imitation, of course, is critical in social learning of goal-directed actions (e.g., Meltzoff, 1995; Carpenter et al., 1998). But, even though it often occurs in interactive contexts, it is important to note that it can also occur outside of interactions, such as in observational, or third-party, learning, which develops as early as 12–18 months of age (e.g., Bandura, 1962; Matheson et al., 2013). So, imitation, as such, is fundamentally unidirectional. Only one of the actors need be aware of the common object, and possibly a common intentional relation, of both actors. Indeed, in a simpler situation, an animal, for instance a monkey, can attend to the object-oriented actions of another animal and not act themselves, though they may attend to the same object, and have some understanding of the other individual’s intentional relation at a sub-personal level through processing by mirror neurons in their premotor cortex (Gallese et al., 1996; Rizzolatti et al., 1996; Barresi and Moore, 2008). Matching here does not depend on any form of reciprocity. Moreover, there are numerous situations where several animals near each other may attend to a suddenly noisy object at the same time, without any awareness that their ‘matched’ intentional relations are co-occurring. Second personal relations to shared objects can only occur when both individuals are aware of each other at the same time as they are sharing a common object of intentional relations. In the typical case, they are also aware that they share a common object of their intentional relations or are in a position to acknowledge to each other in some way that they share this common object of interest.

Intentional relations to shared objects provide a richer form of second-person information than those discussed so far because the shared object provides both an anchor and a pivot for the intentional relations of both interactive partners to interconnect. In this way, alignment of intentional activity toward common or shared objects creates a more stable form of dynamic interdependence between the activities of interacting agents. As we shall later argue, this interdependence yields an ongoing source of second-person information that provides a substrate for knowing both agent and object poles of the intentional relations of both self and other.

### Summary

We have distinguished five aspects of second-person information that are available in the experience of interaction but not from the experience of observing individual actions, either of the self or of others. It should be clear that these second-person forms of information are quite distinct from first- or third-person forms of information because they reflect to some degree how the intentional relations of two or more interacting intentional agents are connected, something that neither first- nor third-person forms of information provide. In consequence, for participants in an interaction, these forms of information potentially provide the information base for bridging the divide between self and other. Yet, the extent to which these aspects of information are only evident to participants in the interaction is debatable. A keen observer of an interaction between two other agents may be able to glean much of this second-person information. Certainly, the simple temporal patterning information of contingency, and to some extent the relevance information of reciprocity as well as matched intentional relations may well be apparent to an observer outside of the interaction. However, we suggest that self-directedness, affective engagement, and shared intentional relations provide forms of second-person information that are peculiar to being a participant in an interaction. It is simply not possible to gain the same kind of access to these forms of information in a direct way without being involved as a participant in an interaction. An external observer may note directions of gaze that ground self-directedness, the emotions expressing affective engagement, and the coordination involved in contingency, reciprocity, and shared intentional relations, but external access to this information is not the same as the second-person experience of it that is available to a participant, where it can play an important and unique role in the development of social understanding.

We suggested earlier that neither first- nor third-person information are sufficient on their own to allow social understanding in terms of a conceptualization of intentional relations as the object-directed psychological activity of persons. How might second-person information help a participant in social interaction reap the benefits of what that interaction has to offer for the development of the understanding of intentional relations? As noted in the subsections above, each of these forms of second-person information has been theorized at different times to be important for the development of social understanding. We have provided some critical analysis of the limitation of certain forms of second-person information for yielding an understanding of intentional relations. In the final section, we take a more positive approach to this enterprise and show how key forms of second-person information are essential for the acquisition of mature human social understanding.

## Pulling it Together

In this section, we assume a developmental approach to answering the question of how second-person information contributes to social understanding. Our position is that mature forms of social understanding are best understood with reference to their developmental origins (see also Barresi and Moore, 1996; Moore, 2006). We further suggest that different forms of second-person information play different roles at different stages of development. Preempting the more detailed account offered below, our story will be simple. The majority of the forms of second-person information outlined above together provide a necessary but not sufficient substrate for the development of social understanding. Self-directedness, contingency, affective engagement, and relevance provide the conditions for reciprocal interaction to become established. But it is only with the advent of shared intentional relations that an understanding of self and others as persons with intentional relations may arise.

### Second-Person Information Prior to Shared Intentional Relations: The Development of I-You Relations

Self-directedness is the fundamental condition from which social interaction grows. Infants have the perceptual sensitivity to discriminate certain forms of self-directedness information, such as direct versus averted eye contact, from birth. Furthermore, they prefer to attend to social information that carries self-directedness information. Perhaps the key example of this is direct eye contact. Other supportive examples are a visual preference for facial configuration over non-faces (Morton and Johnson, 1991) and an auditory preference for infant-directed speech over adult-directed speech (e.g., Cooper and Aslin, 1990). This repertoire of perceptual preferences means that infants orient toward and allocate more attention to stimuli that present such self-directed forms of information. Without such a repertoire of preferences, infants would not be in a position to gain this first form of second-person information and respond to it in a complementary other-directed fashion through attention to its source. As a consequence, this tendency to attend to second-person information in this way means that infants present to others in their world as ready and willing to interact.

Very quickly following on from the primordial state of social attention, infants start to demonstrate actions that enable participation in interaction. Growing motor control allows infants to experience effects in the world that occur contingently on their rudimentary actions. But perhaps the most significant of these actions is smiling. Infants begin to smile within 2 months of birth and critically one of the best elicitors of infant smiling is the full frontal facial configuration of others displaying self-directedness. Contingency is also effective as an elicitor of smiling, such that events that follow the infant’s own action in a reliable manner lead to a so-called ‘mastery’ smile (Watson, 1972). So, within 2 months of life infants are producing social actions – smiles – directly in response to two of the basic forms of second-person information: self-directedness and contingency. Smiling is perhaps the single most important action in establishing social interactive structures. When infants smile, adults feel tremendously rewarded and they work hard to reproduce the expression. Because infants tend to smile toward self-directed and contingent information, caregivers and others react to the production of smiles by providing more of the same – more self-directedness and more contingency. As noted earlier, infants in general actually prefer less than perfect contingency and those most familiar with any particular infant quickly tune into the contingent patterning of stimulation that infant finds most rewarding. Over time, mothers and infants tune into the quality of each other’s stimulation so that their interactions become finely coordinated. Because adults also find these interactions rewarding, they too experience and show positive affect, and so affective engagement ensues as each partner’s positive affect is reflected in the other.

Once infants are showing affective engagement in response to contingent social stimulation, the ground has been fully prepared for reciprocal interaction to occur. Affective engagement is mutually rewarding and ensures that both participants are motivated to maintain their interaction. The first interactions of this sort are commonly termed ‘dyadic’ because they occur between two interactive partners – most typically an infant and the mother – but do not yet involve a shared object or focus. These interactions start out as primarily organized by the adult participant, with the mother essentially inserting her infant-directed action around relatively disorganized infant action (e.g., Kaye, 1979; Schaffer, 1984). However, before long dyadic interactions show evidence of being mutually regulated (Kaye and Fogel, 1980; Tronick et al., 1980; Rochat et al., 1999). In these interactions both participants respond to the actions of the other and they both have expectations of their partner’s action. At this point, the success or failure of the interaction depends upon the relevance of each participant’s action to the other’s action, that is how well each participant’s action serves as a response to the other’s action in order to maintain the mutual engagement.

This brief account of the early development of social interaction shows that four forms of second-person information are critically involved in enabling social interaction during the first 6 months of life. First self-directedness information recruits the infant’s attention and leads to attention to the interactive partner. Second, infant perception of self-directedness information and detection of contingency information leads to infant smiling. Third, infant smiling is matched by the partner’s own smiling, thereby generating affective engagement. Finally, the pleasure generated by these conditions leads to a motivation in each participant to find ways of responding relevantly to the other’s action in order to maintain the interaction in a reciprocal fashion.

### The Rise and Role of Object Interest: The Development of I-It Relations

Awareness of the four forms of second-person information examined in the previous section is developmentally essential for laying the foundation for social understanding. However, to get to an understanding of intentional relations, more is needed. The next step is for infants to acquire a greater interest in the non-social world. Until about 4 months of age, infants find social stimulation particularly appealing. As we have noted, this interest comes from natural preferences for social stimulation, but it also is a result of significant limitations in infants’ visual perceptual, postural, and motor abilities. Social stimulation comes to the infant, so even though infants have very little control over their motor abilities and their visual perception is poor, they can still benefit immensely by the willingness of adults to stimulate them. By about 4 months, infants have gained much greater control over their neck and torso muscles, so that they can sit with support and hold their heads steady. This control enables a further significant ability, visually guided reaching. So now, even though they cannot yet locomote independently, they can look out into the world and reach for objects that attract their interest. Maturing postural and motor control, as well as visual acuity, during the middle of the first year of life leads infants to acquire a growing interest in non-social objects. Toys can be grasped and brought closer for examination, which may initially be largely oral but before long will involve uni- and later bimanual manipulation under inspection. During this period, objects become worthy competitors to people as objects of fascination. The proportion of time that infants spend in face-to-face interactions with people drops considerably by 6 months (Kaye and Fogel, 1980).

Interestingly, the growing preoccupation with non-social objects in infancy is initially a quite solitary affair. Infants will manipulate and examine objects but not offer them to interactive partners. A mother, noticing her infant’s new found interest in objects may facilitate this object interest by presenting toys to her child. But having accepted the toy, the infant will typically examine it, mouth it, shake it, bang it against a surface, before eventually dropping it or pushing it away. What does not happen at this initial stage of object interest is offering the object back to the parent, or holding it up to show to the parent (or anyone else), or otherwise using it to engage the adult in interaction. So at this point in development, infants can engage with people in dyadic interactions (I-You) and they can engage with objects in exploration and play (I-It), but they do not combine the two modes into object-focussed interaction.

Nevertheless, with the growth of infants’ object interest, adults recognize that objects are now an important route to their infants’ hearts, and they incorporate toys into their interactions. Mothers become willing ‘go-fers.’ They will present toys to their infants for examination and then retrieve and return the toy after the infants has dropped it or pushed it away. Initially, infants’ roles in these object-centered events are confined to manipulation, examination, and rejection. But gradually and almost imperceptibly over the next few weeks, the interactions start to become more reciprocal. Rather than simply pushing a toy away, the infant may push it toward the mother. And associated with this development, a profound change in attention occurs. Rather than simply concentrating on the toy, the infant acts on the toy and then looks at the mother’s face, as if anticipating the mother’s reaction and what she will do next. From this point on, object-centered social interactions are characterized by rapid switching of attention between object and person. The infant has now crossed a watershed into a fundamentally new phase of life – triadic interactions – that will characterize essentially all social interaction from this point on.

### Shared Intentional Relations: The Development of I-You-It (or We-It) Relations

Triadic interactions involve infant and mother reciprocally engaged in relation to some object or event that is of mutual interest (Trevarthen and Hubley, 1978). Within triadic interactions there is typically a degree of alignment of attention, of affect, and of purpose between the participants. These interactions are an extension of earlier dyadic interactions but now incorporate an object of mutual attraction. As such they present the form of second-person information that we referred to earlier as shared intentional relations. Both participants have an intentional relation to the object but furthermore these intentional relations are more or less aligned so that each participant experiences their own intentional relation to the object, the other’s intentional relation, and the extent of the alignment between them. Critically, infants are aware of whether or not there is alignment and will begin to regulate the interaction to increase alignment and thereby achieve sharing of intentional relations.

Clear examples of triadic interaction observed by the end of the first year of life include joint visual attention (e.g., Corkum and Moore, 1995), whereby one partner follows or directs the visual attention of the other, in an attempt to achieve alignment of the epistemic intentional relations to some object or event. Social referencing (e.g., Feinman, 1982), whereby the infant acquires an emotional relation to some ambiguous object from observing the emotional display of the mother, represents triadic interaction involving shared affective intentional relations. Object-focussed imitation (e.g., Meltzoff, 1988) shows how goal-directed action may be shared between participants in triadic interaction. In all these cases, infants are exposed to second-person information involving shared intentional relations.

We argue that the second-person information of shared intentional relations that is available in triadic interaction plays a pivotal role in the development of an understanding of intentional relations. Through participation in triadic interactions with shared intentional relations, infants gain second-person experience of the correspondence between their own and the partner’s intentional relation. The gap between the first-person experience of intentional activity and the third-person experience of observing another’s intentional activity can be bridged so that a form of representation of intentional relations that can be applied uniformly to self and other can be formed. It is only through alignment of object-directed intentional relations, and the mutual attention found in triadic interactions that this common form of representation is possible (cf. Moore and Paulus, 2013). In bridging the intentional gap between self and other, it is important to recognize that whereas the gap may appear significant in the case of relatively abstract intentional relations such as beliefs and desires, the gap is particularly short and transparent in the case of manual actions on, or visual attention to, objects that are part of the immediate space of a triadic interaction between mother and infant (Barresi et al., 2013). So, it is these interactive conditions that provide the epistemic substrate for the construction of representations of intentional relations that may ultimately be applied equivalently to both self and other.

In the rest of this section, we concentrate on differentiating the social understanding made possible by the shared intentional relations of triadic interaction both from what precedes it in the dyadic phase of development and from what comes later.

Prior to triadic interaction, we argue that despite the availability of other forms of second-person information, which serve to keep infants motivated and engaged in interaction, infants use distinct forms of representation of intentional relations for self and for other. For their own intentional relations, younger infants understand their own activity primarily in a first-person form. This first-person form of understanding focuses on the object of the intentional activity, with only implicit awareness of the self. For the intentional relations of others, younger infants use a third-person form of understanding for which the object is at best implicit. Whereas some degree of sub-personal integration of first- and third-person information may occur in observing others, perhaps through processes involving mirror neurons (Moore and Barresi, 2009), this direct perception of the activity of others does not depend on I-You relations, so cannot ground a common form of representation that can be applied both to self and other. Yet, without the prior experience of entering into and maintaining I-You relations through processing of earlier forms of second-person information, the triadic form of interactive activity that enables understanding of intentional relations would not be possible. Thus, interaction of the dyadic sort, and the second-person information it provides, is a necessary condition for, but does not by itself constitute, the understanding of intentional relations that arises through triadic interactions.

How then does the understanding of intentional relations made possible by the onset of triadic interaction differ from later forms of social understanding? From our point of view, shared intentional activity at the triadic level is a genuine form of collective intentionality (e.g., Searle, 1990, 1995; Butterfill, 2012; Tomasello, 2014) or ‘we-mode’ (e.g., Gallotti and Frith, 2013). We suggest that the immediate limitation at the onset of triadic interaction is that there is not yet a conception of a distinction between the individual agents or persons – the ‘I’ and the ‘you’ – doing the sharing, nor likewise the conception of ‘you and I’ as a collective ‘we’ that has a shared goal. Infants at this point in development can determine whether or not the individual partners’ object-directed activity is relevant and reciprocal – whether or not there is alignment of the intentional relations – but they do not represent the activity as the joint efforts of two independent intentional agents or persons orienting their attention, sharing an emotional attitude, or attempting to achieve a common goal. That is, while they are aware of the essential equivalence in object-oriented activity of the self and the other engaged in this shared activity, they have not yet formed the appropriate concepts to represent this equivalence, which, on our account comes about through the intentional schema that integrates this equivalence into intentional concepts that take a bivalent form involving both first- and third-personal aspects of intentional relations (Barresi et al., 2013; Musholt, 2015).

It is perhaps worth comparing briefly this account of infants’ collective intentionality with that of some others who have provided analysis of this concept (e.g., Searle, 1990; Bratman, 1999; Gilbert, 2009; phenomenologists). Some (e.g., Bratman, 1999) have argued that collective intentionality rests on some level of meshing of representations of individual intentionality. In contrast, our view is that participating in collective intentionality precedes the representation of individual intentionality (e.g., Barresi and Moore, 1993). In this respect our account characterizes infants’ collective intentionality as closer to the way in which Searle (1990) generally characterizes collective intentionality as an attribute of an irreducible ‘we.’ However, in our account this ‘we’ is understood initially in a non-conceptual form.

We also are skeptical of the idea that collective intentionality at the level of triadic interactions entails a joint commitment to act together (cf., Gilbert, 2009) because this too would require an appreciation of the collective nature of the shared intentional activity. Whether collective intentionality presupposes an appreciation of norms (cf., Gilbert, 2009) is more ambiguous. We suggest that infants do not respond to norms understood as social regularities, however, if one interprets norms to be representations of the regular patterns of interaction previously experienced by infants in interaction with others, then we would agree that triadic interactions presuppose the establishment of such interindividual norms.

The distinction between an undifferentiated or non-individualistic form of collective intentionality emerging with shared intentional relations and a more mature form of collective intentionality that recognizes the equivalence of self and other as independent persons is consistent with evidence that whereas children can learn novel intentional relations in interactive settings by the end of the first year of life (e.g., Meltzoff, 1988), they only readily acquire novel intentional relations in purely observation or third-party setting toward the middle of the second year (e.g., Herold and Akhtar, 2008; Matheson et al., 2013). Furthermore, the ability to acquire novel behaviors in observational contexts is linked to achieving other markers of an individualistic understanding of persons, such as self-recognition (Herold and Akhtar, 2008; Matheson et al., 2013).

Nevertheless, we argue that the shared intentional relations information available from non-individualistic collective intentionality is the critical information for the construction of person-level understanding of intentional relations. In particular, situations in which there is a misalignment of the intentional relations of self and other within triadic interactions may provide particular fertile ground for the differentiation of the intentional relations of self and other from those shared by a collective ‘we.’ It is during the second year of life that infants come to recognize persons as selves and to recognize that selves engage in both independent and collective object-directed intentional activity (Moore, 2007; Moore and Barresi, 2009).

## Conclusion

Second-person approaches to explaining human social understanding offer an essential complement to traditional theory theory and simulation approaches, particularly when it comes to the development of this understanding. Whereas the latter approaches assume that processing of the first- and third-person information available from individuals’ activity is suitable for yielding social understanding, we have argued that by themselves they are insufficient. Instead, social understanding depends fundamentally on social interaction because of the availability within interaction of a number of forms of information that we collectively refer to as second-person information. We distinguish five forms of second-person information that provide the critical substrate for the development of social understanding: self-directedness, contingency, reciprocity, affective engagement, and shared intentional relations. We propose a developmental account that articulates how each form has a role to play in the construction of social understanding.

In closing, we would like to outline three take away messages from this paper. First, the key aspect of the second-person approach is the recognition of forms of information that are fundamentally interactive and not available from the observation of the activity of individual agents, whether self or other. Second-person information is necessarily a property of the interactive activity of two (or more) agents – it reflects the dependence of the activities of the two agents on each other.

Second, within the second-person approach, it is not mainly being a participant in an interaction that is crucial in the development of social understanding, but the fact that the particular forms of information that are generated only in interactions are essential in bridging the gap between self and other and thereby drawing the infant into increasing understanding of the shared intentional activity that occurs in the triadic period and out of which initial understanding of intentional relations is constituted. It is these forms of information that can be used by the infant’s information-processing system both to guide social interaction and to build social understanding. There is no question that being a participant in the interaction makes these forms of information much more salient. Indeed, it is likely that the construction of social understanding through early development would be made much more difficult, if not impossible, without participation in social interaction. But, if the only information that occurred in interactions were the same first-person and third-person forms of activity that occur outside of interactions, mere participation would hold no privileged position in the acquisition of social understanding. It is the difference in the kinds of information available in interactions that is of fundamental importance.

Third, there are multiple forms of second-person information and all play a role in social understanding. No single form of second-person experience is uniquely qualified to generate an understanding of self or others. To focus theoretical attention on one or other form (e.g., self-directedness and affective engagement) is to miss the essential role that all play in the acquisition of social understanding through development.

## Author Contributions

CM and JB jointly planned the article content. CM took the primary role for drafting the manuscript as well as subsequent revision. JB made substantial contributions to the revision of the article as well as drafting some sections.

## Conflict of Interest Statement

The authors declare that the research was conducted in the absence of any commercial or financial relationships that could be construed as a potential conflict of interest.

## Footnotes

1 See Musholt (2015, 2017) for a philosophical analysis of the non-conceptual and non-reflexive understanding of intentional relations that is involved in direct perception and action in interactive contexts in early development, and for the development of conceptual understanding of intentional concepts. A similar distinction appears in the phenomenological tradition in Husserl’s and Merleau-Ponty’s distinction between operative intentionality and act (or thetic) intentionality, the former being non-conceptual and the latter conceptual (cf. Merleau-Ponty, 2012).

2 It is also worth mentioning that Murray and Trevarthen’s (1986) has been criticized for lacking a control period and subsequent attempts at replication and refinement have shown a rather more complex picture of effects than originally claimed (see Muir and Hains, 1999).
